# Long COVID in children and young people: then and now

**DOI:** 10.1097/QCO.0000000000001136

**Published:** 2025-08-13

**Authors:** Anna Coughtrey, Snehal M. Pinto Pereira, Shamez Ladhani, Roz Shafran, Terence Stephenson

**Affiliations:** a UCL GOS Institute of Child Health; b University College London Division of Surgery & Interventional Science; c UK Health Security Agency, London, England, UK

**Keywords:** adolescents, children, COVID-19, long COVID, SARS-CoV-2

## Abstract

**Purpose of review:**

On 11 March 2020, the WHO characterized COVID-19 as a pandemic. A clinical case definition for post-COVID-19 condition in children and adolescents by expert consensus was agreed by the WHO in 2023. It is now 5 years since the WHO declared a pandemic, and this review aims to summarize key advances in our understanding of long COVID over those 5 years.

**Recent findings:**

That symptoms could persist in adults and CYP for months after initial infection was first reported in Autumn 2020. Long COVID in adults is frequently characterized by symptoms of fatigue and breathlessness but brain-fog, joint and muscle pain have been reported much more commonly in adult follow-up than CYP. The most common persisting symptoms experienced by CYP after COVID-19 infection in initial studies, often with less than a year of follow-up, were fatigue, headache, shortness of breath and persisting loss of smell and taste. With longer follow-up, up to 2 years, the commonest symptoms still include not only fatigue, headache and shortness of breath but also sleep difficulties, whereas loss of smell and taste persisted only in a minority. However, many symptoms were almost as common in test-negative controls, raising questions about the causal role of SARS-CoV-2 virus. Predictors of long COVID, as defined, were female sex, history of asthma, allergy problems, learning difficulties at school and family history of ongoing COVID-19 problems.

**Summary:**

The implications of the findings for clinical practice and research are that long COVID is not the same in CYP as adults; both their physical and mental health should be studied; and intervention trials are needed.

## INTRODUCTION

As early as March 2020, patient advocacy groups highlighted that survivors of SARS-CoV-2 were often left with persistent health issues. Similar problems were subsequently described in children and young people (CYP). The terms ’long COVID’ or Post COVID Condition (PCC, as used by the World Health Organization) and Post-Acute Covid Sequelae (PASC, the term used in the United States) were all coined but there was no early definition for long COVID in CYP, unlike in adults for whom the WHO, NICE (UK) and CDC (USA) all produced different definitions. Furthermore, a standard dataset to be collected was not recommended early in the pandemic [1]. As a result of these delays, routine administrative data was not systematically captured in health datasets.

Moreover, early research focused on adults, leaving a gap in understanding long COVID in CYP, particularly as acute COVID-19 illness was generally milder in CYP, although both a ‘Kawasaki-like’ syndrome [2] and an increased risk of myocarditis [3] were described as potentially serious acute complications of SARS-CoV-2 infection in CYP. The physical, social, educational and psychological long-term consequences of COVID-19, however, had the potential to significantly impact the transition of CYP to adulthood. It was crucial to define the clinical characteristics of long COVID in CYP and to identify those most at risk in the hope of developing targeted interventions.

An early systematic review [4] including 23 141 CYP showed a significantly increased risk of persisting cognitive difficulties, headache, loss of smell, sore throat and sore eyes in post-COVID cases compared to controls. Female sex was a risk factor and, perhaps unsurprisingly given the demography of persisting postviral symptoms following Epstein–Barr virus and hepatitis B virus, older CYP had a higher prevalence of all persisting symptoms except cough.

These observations, however, told us nothing about potential underlying mechanisms and only 5 out of the 22 studies had controls to address whether the symptoms reported by CYP might be associated with SARS-CoV-2 infection or potentially influenced by a range of other pandemic-related factors, such as social isolation, anxiety, depression, and educational concerns. Our own research focused on adolescents with confirmed SARS-CoV-2 infection rather than young children, and we used a matched, initially noninfected control group. We assessed both physical and mental health, before and during the pandemic, rather than asking about, at that time, whether they suffered from a poorly defined syndrome termed ’long COVID’ [5]. It transpired that our initial objectives (to agree a definition of long COVID based on the clinical phenotype in CYP and hence report the prevalence) were simple in concept but much more difficult in execution. 

**Box 1:**
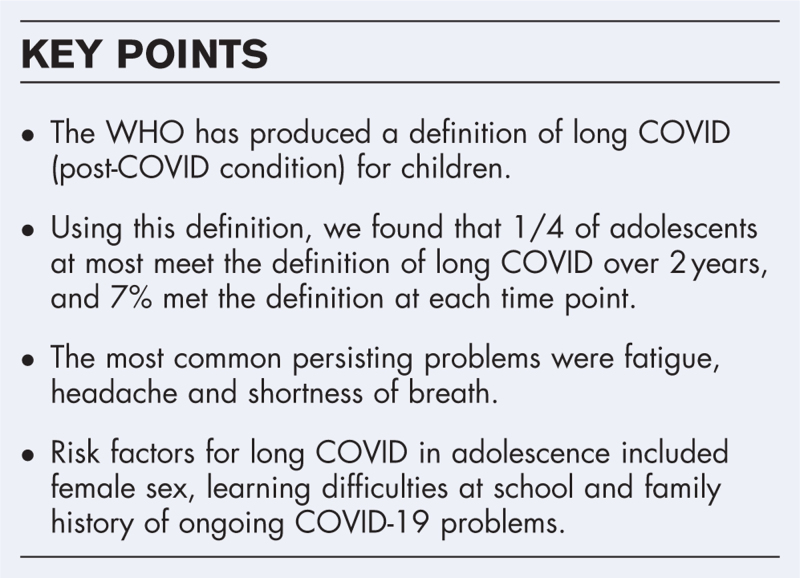
no caption available

## THE CLoCk STUDY

We initiated a longitudinal national cohort study focused on 31 012 CYP aged 11–17 years when they tested positive for SARS-CoV-2 during September 2020 to March 2021. Test-positive CYP were matched to age-matched, sex-matched, and geographically matched SARS-CoV-2-negative peers at study invitation. Participants completed online questionnaires assessing their physical and mental health at 3, 6, 12, and 24 months after their initial positive or negative PCR test.

As rates of infection and re-infection rose with successive waves of wild, alpha, delta and omicron variants, along with termination of community testing, and the introduction of a national COVID vaccination programme, by 2022, over 99% of adolescents in England had SARS-CoV-2 antibodies [6^▪^]. Therefore, we could no longer compare test-positive CYP with uninfected controls. In the latter stages of our research, therefore, we compared ‘initial test-negatives with no subsequent positive test’ (NN); ‘initial test-negatives with a subsequent positive test’ (NP); ‘initial test-positives with no report of subsequent re-infection’ (PN); and ‘initial test-positives with report of subsequent re-infection’ (PP). We also explored the impact of infection or reinfection with the Omicron variant of SARS-CoV-2 infection on CYP 12 months post infection [7].

## RESEARCH DEFINITION OF LONG COVID IN CHILDREN AND YOUNG PEOPLE

The definition we developed using a consensus methodology [8] was ‘A condition in which a child or young person has symptoms (at least one of which is a physical symptom) that:

(1) Have continued or developed after a diagnosis of COVID-19 (confirmed with one or more positive COVID test)
(2) Impact their physical, mental or social wellbeing
(3) Are interfering with some aspect of daily living (e.g. school, work, home or relationships) and
(4) Persist for a minimum duration of 12 weeks after initial testing for COVID-19 (even if symptoms waxed and waned over that period)’.

This is the definition we used in our subsequent publications operationalized [9] as requiring at least one physical symptom, AND experiencing at least some problems or reporting to be very worried in any one of the five domains captured via the EQ-5D-Y scale [10]. A clinical definition was also agreed with the WHO for global use, which was similar but did not insist on laboratory confirmation of SARS-CoV-2 infection and required symptoms lasting at least 2 months and starting within 3 months of confirmed or probable SARS-CoV-2 infection [11^▪^].

## KEY FINDINGS OF THE CLoCk STUDY

Our findings demonstrated that, at 3 months post-PCR testing, the most common persisting symptoms were fatigue, headache, shortness of breath and persisting loss of smell [12]. Apart from loss of smell, these symptoms remained the commonest at 2 years follow-up along with sleeping difficulties [6^▪^]. However, many symptoms were almost as common in test-negative controls, indicating that the symptoms could not solely be attributed to SARS-CoV-2 infection. Importantly, too, mental health of test-positives was similar to test-negatives. At 6 months posttesting, an association between having a parent with ongoing problems after COVID-19 and long COVID in CYP was also noted [13]. Predictors of long COVID up to 24-months postinfection in CYP were female sex, pre-COVID-19 health (in particular, asthma and allergy problems), learning difficulties at school and family history of ongoing COVID-19 problems [9].

The persisting symptoms of long COVID in CYP were similar but not identical to adults in whom cognitive problems and muscle pain were more common than in CYP [14^▪^,^15^].

A systematic review and meta-analysis of 194 studies with 735 006 patients with a mean follow-up of 4 months [16] found the prevalence among nonhospitalized adults was 34% for fatigue, 20% for breathlessness, 17% for muscle pain, 15% for sleep disorders, 12% for smell dysfunction and 8% for taste dysfunction.

From a sub-cohort who responded at all four time points (3, 6, 12 and 24 months post-PCR test), only 7% of our original participants who received a positive index PCR test met the research definition for long COVID persistently at all follow-up time points up to 24 months [6^▪^]. Hence, over time, the prevalence of those persistently meeting the long COVID definition declined, such that approximately 75% of CYP with long COVID at 3 months after infection no longer fulfilled the definition of long COVID at 24-month follow-up. However, new symptoms emerged in other individuals at later stages of follow-up [14^▪^]. Repeated cross-sectional surveys published in other reports may, therefore, have given misleading information about the persistence of symptoms. Our longitudinal methodology using ‘within person’ follow-up showed unequivocally that symptoms declined with time in most CYP. With an increasing interval between initial infection and the emergence of new symptoms, it is less likely that symptoms emerging later were causally related to the initial SARS-CoV-2 infection, although of course herpes viruses can cause new symptoms many years after initial infection. However, we did find evidence of a relationship between number of infections and likelihood of persisting symptoms, consistent with criteria for causality [15]. Findings from the study of those infected with the Omicron variant were similar to those of the main CLoCk study, and it was concluded that clinicians may not necessarily need to consider number of infections and type of variant when developing treatment plans [7].

Of 576 CYP who received a COVID-19 vaccine after having previously tested PCR-positive for SARS-CoV-2 infection, the proportion meeting the definition of long COVID 6 months posttest was similar to those not vaccinated. So vaccination postinfection did not appear to mitigate the risk of long COVID in CYP already exposed to SARS-CoV-2 [19]. In contrast, CYP who received a COVID-19 vaccine followed by weekly SARS-CoV-2 testing were less likely to develop long COVID during the Omicron variant wave [20]. An Italian observational study also showed that COVID-19 vaccination was associated with a significantly lower risk of adolescents developing long-COVID at 3, 6 and 12 months post-infection [21].

## LIMITATIONS OF THE CLoCk STUDY

The overall response rate for the CLoCk study was 14.1%, with slightly higher participation among female individuals, older adolescents and less deprived CYP compared to those initially invited. This response rate is similar to other random sampling studies, including online surveys, without a previous patient relationship. For example, the response rate to the Office of National statistics COVID-19 Infection Survey when using random households from 13 July 2020 was 13% [16]. The demographic characteristics of test-positive and test-negative participants showed minimal differences, reflecting the matched cohort study design. We recognize the possibility of misclassification, with some CYP potentially being incorrectly identified as SARS-CoV-2 negative or positive, particularly with the cessation of free community testing in April 2022. The study design may also have introduced selection bias, as it could favour participants with online internet access and CYP with symptoms may have been more inclined to participate. Further waves of COVID-19 variants in combination with the cessation of community testing in April 2022 rendered the test-negative controls less certain. Hence, the CLoCk study was simple in concept but much more difficult in execution because of the gradual loss of true negative controls. For that reason, we also decided to examine prepandemic symptom prevalence in CYP for comparison.

## PRE-PANDEMIC SYMPTOM PREVALENCE

The results from the systematic review of CYP and from the CLoCk findings indicated that young people experience a wide range and diverse set of symptoms following SARS-CoV-2 infection, many of which are nonspecific. It is, therefore, essential to understand how the prevalence of these symptoms compare to the prepandemic health of CYP. We conducted a systematic review of the literature to identify studies of symptom prevalence in children aged 10–19 years before the COVID-19 pandemic. We identified 26 sources during 1986–2019 that varied widely in methodology and findings. Overall, there was a high prepandemic median prevalence of cough (13.6%), headache (29.9%) and fatigue (20.5%) [17]. Moreover, between 2012 and 2013 and 2021 and 2022, annual hospitalizations for mental health concerns increased by 65% in England (and this increase preceded the pandemic), whereas all-cause admissions increased 10% [18].

## REPEAT SYSTEMATIC REVIEW

In collaboration with the WHO, we repeated our original systematic review [19]. A total of 60 studies were included in the updated systematic review involving 333 472 CYP and median follow-up of 3 months after SARS-CoV-2 infection. The longest follow-up at that time was 2 years. Twenty-two (37%) of the 60 studies included a control group, of which, 12 (20%) had sufficient comparative data to include in a meta-analysis [20]. Examining all 60 studies, we found that somatic or constitutional symptoms such as fatigue (47%) and headache (35%) were amongst the most commonly reported symptoms in CYP post-COVID. However, when including controlled studies, which accounted for the high background prevalence of such symptoms in noninfected CYP, we found that the excess in cases over controls was much lower, for example, 5% for headache and 7% for fatigue. These findings suggest that persistent symptoms may occur after COVID-19 in CYP but prevalence is much lower than suggested by many lower quality, uncontrolled studies. Postinfection fatigue has also been reported after other human coronaviruses such as Middle East Respiratory Syndrome (MERS) and severe acute respiratory syndrome (SARS) as well as Epstein–Barr, Dengue, Zika, Ebola and Chikungunya viruses.

## POTENTIAL MECHANISMS OF LONG COVID IN CHILDREN

In a previous publication in this journal, we speculated on potential pathophysiological mechanisms to explain long COVID (see Table 1 reproduced below) [21]. We postulated that persisting viral infection, an abnormal immune response, persisting organ damage, and a combination of social distancing and school closure could all be possible mechanisms, not mutually exclusive. What is the evidence now to support any of these? [22].

**Table 1:** Four potential mechanisms that have been postulated to explain long COVID

1. Damage occurs to organs during acute infection and some of the damage persists leading to long-term symptoms. For example, the ‘CoverScan’ study in adults showed persisting multiorgan impairment in adults. Relatively small numbers of children have been admitted to intensive care with severe organ damage as a result of acute SARS-COV-2 infection. The majority of those with paediatric inflammatory multisystem syndrome temporally associated with SARS-CoV-2 appear to be making a full recovery. Therefore, this mechanism seems unlikely to explain persisting post-COVID syndrome in children and young people.
2. The virus persists in the body, and this leads to the persisting symptoms. This would be analogous to the mechanism of persisting or recurrent symptoms from double-stranded DNA herpes viruses causing recurrent genital and labial herpes and shingles. There has been a case report of biopsy-proven persistence of the nucleocapsid protein from the SARS-COV-2 RNA virus in the gut lining of a child with persisting gut symptoms. In autopsies on 44 adults with COVID-19, SARS-CoV-2 was widely distributed, even among patients who died with asymptomatic to mild COVID-19. Persistent SARS-CoV-2 RNA was detected in multiple anatomic sites, including the brain, for up to 230 days following symptom onset.
3. There is an underlying autoimmune mechanism whereby antibodies raised against the virus cross react against host tissues. This could be analogous to the mechanism of ataxia some weeks after a varicella infection due to an autoimmune inflammation of the cerebellum. We are not aware of any studies supporting this mechanism in post-COVID syndrome in children and young people, although autoimmunity has been suggested as a factor in adults.
4. Finally, perhaps none of the above biological mechanisms explain long COVID. The persistence of symptoms is unexplained as in many postviral syndromes in children.

The evidence regarding potential pathophysiological mechanisms for long COVID in CYP suggests they are similar to those for adults, with the exception of persisting organ damage, which seems unlikely to be a major cause in CYP. For example, only 259 CYP were admitted to paediatric intensive care in the first year of the pandemic in England, which has a population of about 11 million CYP [23^▪▪^]. SARS-CoV-2, or parts of the virus, can persist for months in the tissues of children, and long COVID may be associated with hyperinflammation [24^▪▪^]. This hyperinflammation in some CYP with long COVID can manifest as persistence of the coronavirus spike protein in the bloodstream and altered immune responses involving neutrophils, lymphocytes and cytokines [25]. Whilst endothelial inflammation and microthrombi have been proposed in adult long COVID, there is little evidence for these in CYP although clotting abnormalities have been described in children [26]. The lack of a single, precise mechanisms underlying long COVID and the multiplicity of presenting symptoms suggests the condition will be best managed by a multidisciplinary team with prior expertise of unexplained persistent physical symptoms.

## CONCLUSION

Despite the challenges of researching long COVID in the context of a global crisis of unprecedented scale and impact, our pioneering research in long COVID in CYP has provided policy makers, clinicians, CYP and their families with crucial novel data on the nature and natural course of symptoms of long COVID in CYP. The most common persisting symptoms experienced by CYP after COVID-19 infection were fatigue, headache, shortness of breath and persisting loss of smell and taste. However, many symptoms of long COVID (apart from loss of smell and taste) were almost as common in test-negative controls, raising important questions about the role and contribution of SARS-CoV-2 infection compared to other potential causes, including the effects of lockdowns, school closures and social isolations, sometimes termed ‘long pandemic’. In terms of mental health, we found initially little difference in test-positives compared to test-negatives. Others have described a higher likelihood of developing new mental health disorders in both COVID-19 positive and negative adolescents compared to younger children [27]. There is a continuing need for further research to understand the relationship between long COVID in adolescence and future physical and mental health, education and employment in adulthood. Potentially, data linkage to other relevant national datasets could be useful.

Predictors of long COVID in CYP were female sex, history of asthma and allergy problems, learning difficulties at school and family history of ongoing COVID-19 problems. Vaccines help reduce the risk of COVID-19 and, hence, will also reduce the risk of long COVID, but there is little evidence in CYP that vaccines ameliorate persistent symptoms after COVID. Symptoms declined with time in most CYP, irrespective of the responsible variant or COVID-19 vaccination status, although new symptoms emerged in different CYP, so cross-sectional studies in any population may lead to erroneous conclusions. Research has helped clinicians to inform patients and their carers on the likely natural course of this condition. Future research is needed to further understand the pathophysiology of long COVID in CYP, develop diagnostic tests and identify effective interventions for young people who continue to be significantly impaired by PCC [28].

The lesson for future pandemics is the old message that ‘children are not small adults’ [29]. Long COVID is not the same in CYP as adults, both their physical and mental health should be studied, and intervention trials need to start faster in parallel with adult trials, rather than waiting years for adult trials to report before studying children.

## Acknowledgements

*All authors contributed substantially to the review including conceptualization, drafting and reviewing of the manuscript.*

### Financial support and sponsorship

*Funding: this work is independent research jointly funded by the National Institute for Health and Care Research (NIHR) and UK Research and Innovation (UKRI) [Children and young people with Long COVID (CLoCk) study, Reference COV-LT-0022]. All research at Great Ormond Street Hospital NHS Foundation Trust and UCL Great Ormond Street Institute of Child Health is made possible by the NIHR Great Ormond Street Hospital Biomedical Research Centre. S.M.P.P. was supported by a UK Medical Research Council Senior Nonclinical fellowship (ref: MR/Y009398/1). The views expressed are those of the authors and not necessarily those of the NHS, the NIHR, UKRI or the Department of Health and Social Care.*

### Conflicts of interest

*T.S. is Chair of the Health Research Authority and therefore recused himself from the Research Ethics Application. R.S. co-authored a book published in August 2020, titled Oxford Guide to Brief and Low Intensity Interventions for Children and Young People. All remaining authors have no conflicts of interest.*

## REFERENCES AND RECOMMENDED READING

Papers of particular interest, published within the annual period of review, have been highlighted as:

▪ of special interest
▪▪ of outstanding interest
